# Distinct Metabolomic and Lipoprotein Signatures in Gall Bladder Cancer Patients of Black African Ancestry

**DOI:** 10.3390/cancers17172925

**Published:** 2025-09-06

**Authors:** John Devar, Nnenna Elebo, Ashna Makan, Ariel Pincus, Nicola Lahoud, Stefano Cacciatore, Geoffrey Candy, Martin Smith, Ekene Emmanuel Nweke

**Affiliations:** 1 Department of Surgery, Faculty of Health Sciences, University of the Witwatersrand, Johannesburg 2193, South Africa; john.devar@wits.ac.za (J.D.); nnenna.elebo@wits.ac.za (N.E.); ariel.pincus@students.wits.ac.za (A.P.); nicola.lahoud@wits.ac.za (N.L.); geoffrey.candy@wits.ac.za (G.C.); martin.smith@wits.ac.za (M.S.); 2 Hepatopancreatobiliary Unit, Department of Surgery, Chris Hani-Baragwanath Academic Hospital, Soweto, Johannesburg 1864, South Africa; 3 Bioinformatics Unit, International Centre for Genetic Engineering and Biotechnology, Observatory, Cape Town 7925, South Africa; stefano.cacciatore@icgeb.org; 4 Wits Donald Gordon Medical Centre, Johannesburg 2193, South Africa; ashna.m17@gmail.com; 5 Department of Life and Consumer Sciences, College of Agriculture and Environmental Sciences, University of South Africa, Florida, Roodepoort 1709, South Africa

**Keywords:** gall bladder cancer, gallstones, metabolomics, lipoproteins, cholelithiasis, African patients, LpX, obstructive jaundice

## Abstract

Background: Gall bladder cancer (GBC) is the most common biliary tract malignancy and is often diagnosed at advanced stages, partly due to the absence of reliable biomarkers and limited understanding of its biology in African populations. This study aimed to characterize the metabolomic and lipoprotein profiles of GBC patients of Black African ancestry. Methods: NMR spectroscopy was used to profile the serum samples. Group comparisons used Wilcoxon tests, correlations used Spearman’s rank test, unsupervised analysis was carried out using the KODAMA algorithm, partial least squares modeling estimated free cholesterol (FC) to cholesterol ester (CE) ratios, while multivariate logistic regression evaluated independent predictors. Results: GBC patients showed altered ethanol levels and dysregulated lipoproteins, including increased IDL-C, IDL-TG, and LDL-TG, and decreased HDL-C, HDL-P, and medium HDL-P. Total and conjugated bilirubin strongly correlated with lipoproteins. Unsupervised analysis revealed a GBC subgroup with abnormal lipoprotein profiles and elevated FC/CE ratios, suggesting cholestasis-related LpX formation. Elevated asparagine, reduced ethanol, and an inflammatory metabolic signature characterized the GBC fingerprint. Ethanol and bilirubin emerged as independent predictors of GBC. Conclusions: GBC patients exhibit distinct metabolomic and lipoprotein alterations that may underlie disease progression and serve as potential biomarkers. These findings enhance understanding of GBC pathophysiology in African populations and may inform future diagnostic strategies.

## Simple Summary

Gall bladder cancer (GBC) is the most common cancer in the bile ducts and is often diagnosed late, making treatment challenging. Early detection is difficult, especially in Black African patients, due to a lack of reliable markers. This study looked at metabolites and lipoproteins in blood samples from people with GBC and controls. We showed the metabolic and lipid changes in GBC, which could help explain disease etiology and guide future approaches for early detection and targeted treatment.

## 1. Introduction

Gall bladder cancer (GBC) is the most prevalent cancer of the biliary tract, accounting for 80–95% of cases, and the overall prognosis remains poor, with a 5-year survival of less than 5–10% [1]. Over 80% of these patients are diagnosed at an advanced stage. Worldwide, GBC is the 22nd most common and 17th most deadly cancer [1]. The incidence of GBC worldwide has a geographically variable pattern, with the highest incidence reported in India, Asia, and South America [2]. Although epidemiological studies from African countries, including South Africa, Nigeria, Kenya, and Uganda, suggest that the rate of gallstone disease is low in the Black African population [3], recent studies have demonstrated high rates of incidental GBC in sub-Saharan Africa [4]. Clinical presentations of GBC include loss of appetite, nausea, upper right quadrant abdominal pain, jaundice, and weight loss. However, these are non-specific to GBC and are usually observed at the advanced stage of the disease. About 85% of people who develop GBC have cholelithiasis [5]. Other risk factors include age, sex, gallstones, cholecystitis, family history, and genetic factors. Surgery remains the most effective treatment option, especially in the early stages. However, GBC may recur in approximately 60–70% of patients after surgery, resulting in an unsatisfactory prognosis, with a 5-year survival rate of only 5–15% [6,7].

Numerous studies have been conducted on other ethnicities across diverse geographical regions to gain insight into the development and progression of GBC [8,9,10]. Hence, further investigations are needed on GBC patients of Black African descent [11]. Metabolomic assays on Black African patients have only recently been conducted for specific diseases [12,13,14]. Metabolite concentrations in the blood may reflect the metabolic adaptation of the tumor and could serve as potential biomarkers [15,16]. Nuclear magnetic resonance (NMR) spectroscopy has been used to identify metabolites that can differentiate GBCs from benign cases such as gallstones [17]. For instance, a metabolomics study on Indian patients showed lower concentrations of alanine, creatinine, tyrosine, and branched-chain amino acids in the GBC group. In contrast, elevated pyruvate, glutamate, and formate concentrations were observed in GBC compared to benign gallstone diseases [18]. Furthermore, multiple studies have demonstrated an association between gallstone formation and abnormalities in serum lipid levels [19,20].

In this study, we conducted an untargeted metabolomic profiling of GBC patients of Black African ancestry to identify altered metabolites and lipoproteins that could be linked to the disease in our patient group.

## 2. Materials and Methods

### 2.1. Patient Recruitment and Sample Collection

Ethics approval was obtained from the University of the Witwatersrand Human Research Ethics Committee (Medical) (M230780 and M160640). The study site was the Hepatopancreatobiliary Unit at Chris Hani Baragwanath Academic Hospital, Soweto, Johannesburg, South Africa. Clinically and histologically proven GBC patients aged 18 years and above, self-reporting Black African ancestry, were included in this study. All GBC patients presented at advanced stages (stage III or IV). Our study specifically avoided the inclusion of patients with cirrhosis, as a significant number of these patients harbor a silent hepatocellular carcinoma [21,22] or even a concomitant silent cholangiocarcinoma [23,24]. Benign biliary pathologies (BBPs) were also recruited from the same hospital for the control group. Participants were recruited between January 2019 and December 2020 and provided written informed consent. Demographic and clinical data were captured in the REDCap^®^ database. After collection, blood samples were gently mixed by inverting the tube 3 to 5 times and stored upright at 4 °C until centrifugation.

### 2.2. Sample Processing

Serum was obtained by centrifuging blood samples collected by venepuncture in vacutainer tubes (BD Biosciences, Franklin Lakes, NJ, USA) without coagulant at 1734× *g*, 4 °C for 10 min after allowing it to clot for 30–60 min at room temperature. Serum samples were processed within 2 h of the blood collection. The serum was aliquoted into microfuge tubes (500 µL) and stored at −80 °C until analysis.

### 2.3. Sample Preparation

About 300 µL of thawed serum was added to a solution consisting of 0.75 M potassium phosphate buffer (pH 7.4), 5.81 mM trimethylsilyl-2,2,3,3-tetradeuteropropionic acid (TSP; Sigma-Aldrich, St Louis, MO, USA), and a trace amount of sodium azide (65 mg dissolved in deuterium oxide) to prevent bacterial growth. After vortexing, 540 µL of this working solution was transferred to a 5 mm NMR tube (Wilmad Lab Glass, Vineland, NJ, USA) for analysis. Sample preparation and analysis were performed at the Centre for Human Metabolomics, Potchefstroom Campus, North-West University, South Africa [14].

### 2.4. Nuclear Magnetic Resonance Analysis

The NMR tubes containing the respective samples were loaded on a 500 MHz Bruker Avance III HD NMR spectrometer (Bruker, Billerica, MA, USA) equipped with a triple-resonance inverse ^1^H probe head and x, y, z gradient coils to acquire one-dimensional proton (^1^H)-NMR spectra. Standard nuclear Overhauser effect spectroscopy (NOESY) and a standard diffusion-edited (DIFF) experiment were used to detect signals from small metabolites and high-molecular-weight macromolecules like lipoproteins. Pooled GBC samples were used as a quality control sample and included in each batch for qualitative repeatability assessment by overlaying the raw spectra.

### 2.5. Nuclear Magnetic Resonance Profiling

NMR spectroscopy was used to quantify signals from the samples, which were subsequently identified and quantified. The peaks of the identified metabolites were fitted by combining a local baseline and Voigt functions based on the multiplicity of the NMR signal. The root-mean-square deviation was determined to validate the efficacy of the different deconvolution models. The absolute concentration of each metabolite was calculated according to a previously reported equation [25]. The number of protons contributing to the unknown signals was imputed as 1. The concentration of carbohydrates was also estimated by considering the equilibrium between their cyclic forms.

GlycA and GlycB signals were quantified by integrating the areas between 2.00 and 2.05 ppm and 2.09 and 2.05 ppm, respectively. These signals are indicative of post-translational modifications of glycosylated acute-phase proteins released during inflammation [26]. Lipoproteins were profiled using the Liposcale test (Biosfer TesLab, Reus, Spain). Briefly, the methyl signal of 2D ^1^H-NMR spectra was deconvoluted with Lorentzian functions to quantify the particle number (P) of 9 subclasses, corresponding to large, medium, and small sizes (Z), of the main lipoprotein classes, including: high-density (HDL), low-density (LDL), and very low-density lipoprotein (VLDL) [27]. Cholesterol (C) and triglyceride (TG) contents were quantified for HDL, LDL, and VLDL and also intermediate-density lipoprotein (IDL). The lipid volumes were determined using common conversion factors [28,29]. The assay was performed as previously described [13,14].

### 2.6. Statistics and Data Analysis

Statistical analysis and graphical illustrations of the data were generated in R (version 4.3.2) and R Studio (version 2023.9.0.463) software using in-house scripts. Wilcoxon and rank-sum tests were used to compare differences in numerical covariates (e.g., age and metabolite concentration). Fisher’s exact test assessed differences between categorical variables, and Spearman’s rank test was then used to calculate the correlation coefficient (rho) between variables. The discriminant ability was evaluated using receiver operating characteristic (ROC) curve analysis, and the area under the curve (AUC) was calculated to assess overall model performance. A multivariate logistic regression analysis was performed to identify independent predictors of the pathology using a generalized linear model with a binomial family: odds ratios (OR) with 95% confidence intervals (95%CI) and *p*-values were computed. A *p*-value < 0.05 was considered significant, and a false discovery rate (FDR) of <10% was applied to account for multiple testing.

The KODAMA algorithm, which allows for unsupervised extraction of features and enables analysis of noisy datasets of high dimension, was used to identify patterns representing underlying metabolic phenotypes in all samples [30,31,32]. A training set of DIFF spectra from samples of patients with pancreatic cancer and associated information on the ratio of free cholesterol (FC) to cholesterol ester (CE), retrieved from the study by Elebo et al. [14], was then used to build a partial least squares (PLS) model, as described in [13]. The PLS model was then applied to the DIFF spectra of samples from GBC patients.

## 3. Results

### 3.1. Clinicopathological Features of Gall Bladder Cancer and Benign Biliary Pathology Patients

In total, 40 GBC patients, including 24 patients with stage III disease and 16 patients with stage IV disease, and 27 BBP patients were recruited. The clinicopathological features of the patients with GBC and BBP are reported in Table 1. GBC was shown to be prevalent in older people and females compared to BBP patients.

As expected, GBC displayed elevated bilirubin values compared to the BBP group. GBC patients had also elevated levels of alkaline phosphatase (ALP) and gamma-glutamyl transferase (GGT), although the latter was not significant when compared to the BBP patients. Furthermore, the GBC patients had elevated levels of C-reactive protein (CRP) compared to the controls, which might be due to high levels of cholangitis. Although these clinical parameters reflect the tumor’s impact on liver and biliary function, systemic inflammation, and overall disease burden, no statistically significant differences were observed between GBC patients with stage III and stage IV disease. All GBC patients exhibited elevated bilirubin levels (total bilirubin ≥ 4 µMol/L, conjugated bilirubin ≥ 2 µMol/L). No differences in these markers were noted between GBC patients with and without obstructive jaundice, except for a higher level of ALP in those without obstructive jaundice.

### 3.2. Dysregulated Metabolites and Lipoproteins in Gall Bladder Cancer Patients

Lipid content and lipoprotein particle size data were combined to determine the particle number of each lipoprotein subclass (Table 2). Lipoprotein parameters such as IDL-C (*p* = 0.004, FDR = 0.017), IDL-TG (*p* = 0.003, FDR = 0.017), and LDL-TG (*p* = 0.002, FDR = 0.015) were increased in GBC, while HDL-P (*p* = 0.001, FDR = 0.017) and HDL-Z (*p* = 0.002, FDR = 0.015) decreased significantly when compared to the BBP group.

Metabolites, lipids, proteins, and inflammatory markers (GlycA and GlycB) were shown in Table 3, revealing significantly elevated levels of ethanol concentration in GBC patients when compared with the BBP group.

**Figure 1:**
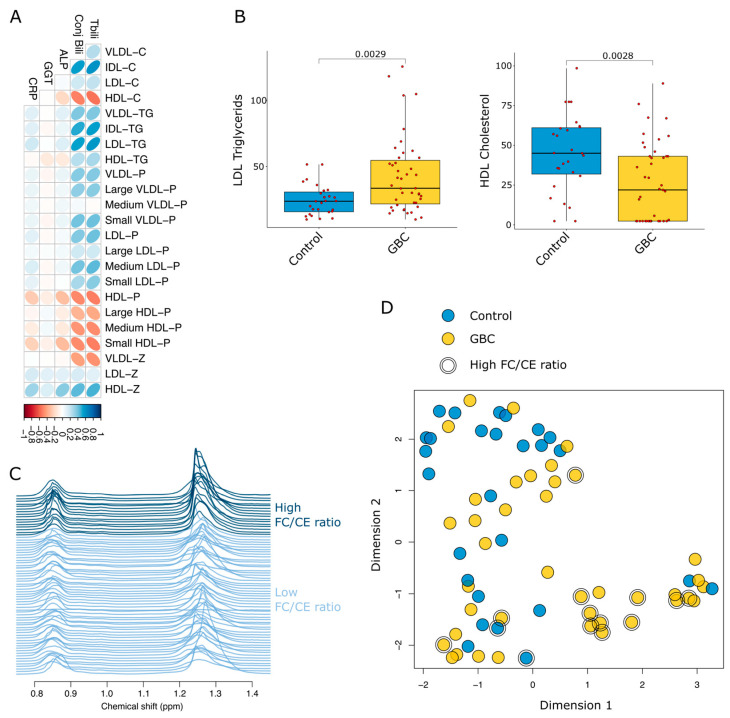
Overview of the lipoprotein profiles in gall bladder cancer and benign biliary pathology patients. (**A**) Correlation between lipoprotein features and clinical parameters. Total and conjugated bilirubin have a strong negative or positive association with lipoproteins. (**B**) Box and Whisker plot comparing LDL, triglycerides, and HDL cholesterol for GBC and controls. (**C**) NMR spectral area of lipoprotein comparing high and low FC/CE ratios. The dark blue lines indicate the high ratios; light blue lines indicate the lower ratios. (**D**) Unsupervised analysis using KODAMA showed that the GBC group in the lower right quadrant has a different NMR profile from others, which may be linked to cholestasis. Abbreviations: ALP, alkaline phosphatase; C, cholesterol; CE, cholesterol ester; Conj Bili, conjugated bilirubin; CRP, C-reactive protein; FC, free cholesterol; GBC, gall bladder cancer; GGT, gamma-glutamyl transferase; HDL, high-density lipoprotein; LDL, low-density lipoprotein; P, particle; Tbili, total bilirubin; ppm, parts per million; TG, triglycerides; VLDL, very-low-density lipoprotein.

Correlation between lipoproteins and clinical parameters showed that total and conjugated bilirubin have a strong association with lipoproteins, as highlighted in Figure 1A. The concentrations of LDL, VLDL and their components were positively correlated with bilirubin levels, whereas HDL concentrations decreased as bilirubin increased. Interestingly, although the size of HDL particles increased, total HDL-C levels declined with rising bilirubin levels. This association was further confirmed when comparing LDL-T and HDL-C levels between controls and patients with GBC (Figure 1B). To investigate whether the observed lipoprotein changes result from bile duct occlusion and the consequent increase in abnormal lipoprotein X (LpX), the FC/CE ratio (a marker of LpX) was estimated from the DIFF spectra. The NMR spectral area of lipoproteins of samples with high and low FC/CE ratios is shown in Figure 1C. The unsupervised analysis of the lipoprotein profiles showed a clear separation of the samples predicted with a high FC/CE ratio (Figure 1D).

**Figure 2:**
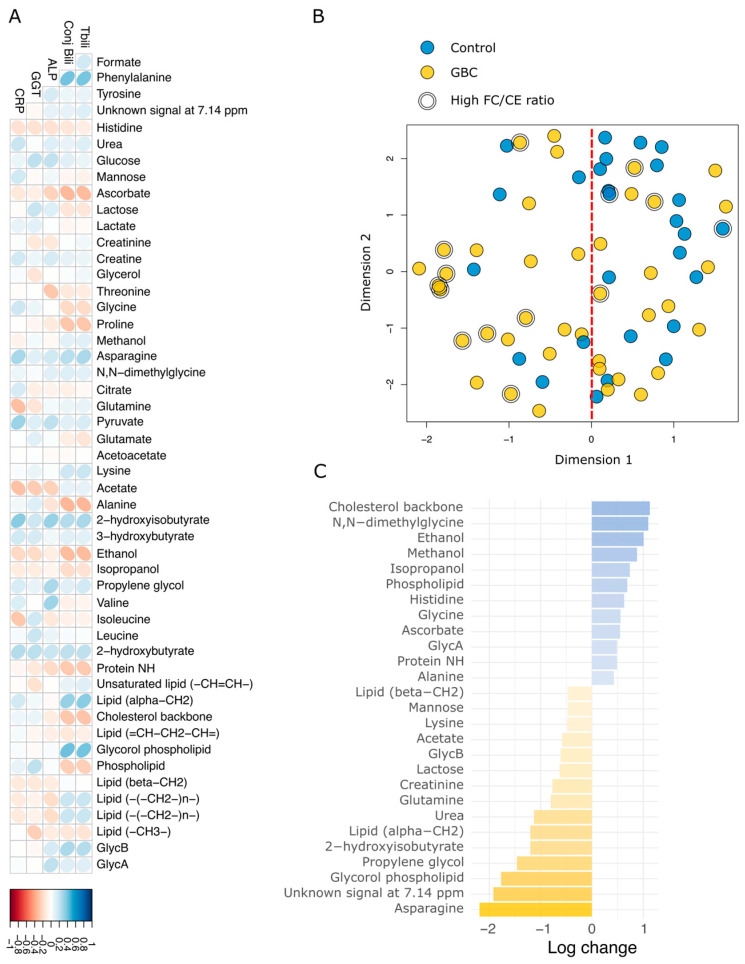
Overview of the metabolic phenotypes in GBC: (**A**) Correlation between metabolic profiles and clinical parameters. There was no strong correlation observed. (**B**) Unsupervised analysis using KODAMA showed little difference between the controls and GBC. However, a larger number of the controls are on the right side. (**C**) Log change of the association of these metabolites with high (blue) and low (yellow) FC/CE ratios showed metabolites with strong positive/negative correlation with high and low FC/CE ratios. Abbreviations: ALP, alkaline phosphatase; CE, cholesterol ester; Conj Bili, conjugated bilirubin; CRP, C-reactive protein; FC, free cholesterol; GBC, gall bladder cancer; GGT, gamma-glutamyl transferase; Tbili, total bilirubin.

**Figure 3:**
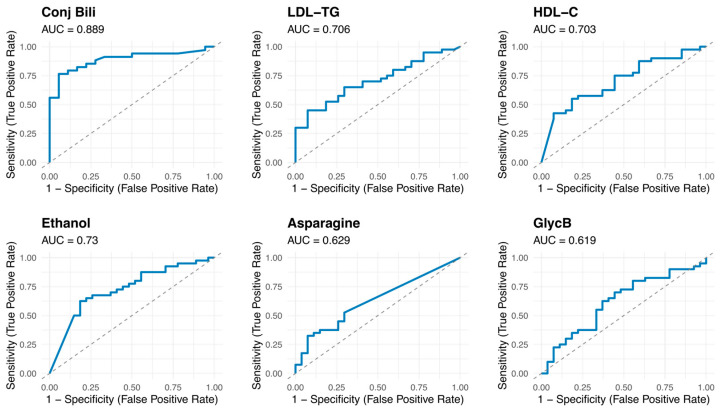
ROC curves for key biomarkers: ROC curves evaluating the discriminative performance of selected markers and their AUC value. Abbreviations: AUC, area under the curve; C, cholesterol; Conj Bili, conjugated bilirubin; HDL, high-density lipoprotein; LDL, Low-density lipoprotein; ROC, receiver operating characteristic; TG, triglycerides.

No strong correlation was observed between the metabolites and clinical parameters (Figure 2A). The unsupervised analysis of the metabolic profiles showed a mild separation between patients with GBC and controls. Leveraging the first dimension of the unsupervised analysis as the primary discriminant, we identified a pronounced asymmetric separation between GBC patients and controls, with 74% of controls clustering on the right side (Figure 2B). The “drivers” of this separation between samples on the left and the right sides are highlighted by the fold change analysis in Figure 2C. Inflammatory markers such as reduced levels of histidine and higher levels of GlycB are associated with the left side, where the presence of samples from patients with GBC is higher (76%). Asparagine, an amino acid essential for cancer growth and development [33], is also increased in patients with GBC. The discriminative ability of conjugated bilirubin, and some altered lipoproteins and metabolites, such as LDL-TG, HDL-C, ethanol, asparagine and GlycB, was then evaluated using ROC curves (Figure 3).

A multivariate logistic regression model was fitted to evaluate the independent association of biochemical, lipoprotein, and metabolic variables with the pathology (Table 4). Among the selected variables included, conjugated bilirubin emerged as a significant predictor of GBC. In contrast, ethanol showed a significant negative association with GBC. In this cohort, conjugated bilirubin and ethanol are independent predictors of GBC.

**Table 4:** Multivariate logistic regression analysis of independent predictors of the pathology.

Features	OR (95%CI)	*p*-Value
Conj Bili	1.03 (1.01 1.06)	0.272
LDL-TG	1.00 (0.94 1.08)	0.006
HDL-C	1.03 (0.98 1.08)	0.253
Ethanol	0.23 (0.05 0.74)	0.032
Asparagine	-	0.854
GlycB	1.41 (0.66 2.88)	0.330

The estimated OR for asparagine was not meaningful, probably reflecting convergence issues related to the large proportion of zero measurements, which can result in unstable logistic regression coefficients. Abbreviations: C, cholesterol; Conj Bili, conjugated bilirubin; HDL, high-density lipoprotein; LDL, Low-density lipoprotein; OR, odds ratio; TG, triglycerides.

## 4. Discussion

This study presents a comprehensive metabolomic and lipidomic profiling of GBC in individuals of Black African ancestry, revealing significant alterations in metabolomic and lipidomic markers associated with disease status and linked to key biological processes. Elevated bilirubin and ALP levels are consistent with cholestasis and biliary obstruction commonly seen in advanced-stage GBC patients, supporting their clinical use as indicators of the disease [34]. Also, elevated CRP levels in GBC patients reflect an underlying systemic inflammatory response, a pattern consistently reported in previous GBC studies [35,36] as well as other malignancies [37,38,39], indicating that this is a general marker of cancer-related inflammation rather than a GBC-specific signature.

Notably, this study identified significantly increased concentrations of IDL-C, IDL-TG, and LDL-TG in GBC patients. These findings suggest that an accumulation of atherogenic lipoproteins and triglycerides may be linked to cancer metabolism [40]. Elevated IDL and LDL components could reflect altered lipid transport and energy metabolism, potentially supporting the enhanced proliferative and survival demands of malignant cells [41]. Furthermore, dysregulated lipoprotein profiles, such as elevated LDL or IDL and decreased HDL, reflect a pro-tumorigenic cholesterol environment. Cholesterol esters stored in lipid droplets support rapid tumor growth and metastasis [42]. High levels of IDL-TG and LDL-TG contribute to a lipid-rich tumor microenvironment, characterized by increased uptake of lipoproteins via LDL receptors on cancer and stromal cells [43]. Conversely, a significant reduction in HDL-C and various HDL particle subtypes (total HDL-P, medium HDL-P, and small HDL-P) was observed in GBC cases. The reduction in HDL particles suggests impaired reverse cholesterol transport, which could contribute to lipid dysregulation in the tumor microenvironment [44].

Importantly, GBC patients had a high ratio of FC/CE, indicative of elevated LpX levels. LpX is an abnormal lipoprotein that accumulates in patients with cholestasis, particularly in those with obstructive jaundice [45]. It is characterized by its unique composition, rich in phospholipids and free cholesterol but deficient in triglycerides and apolipoproteins [46]. Elevated levels of Lp-X have been associated with more severe cholestasis, increased hepatic dysfunction, and poorer overall survival in patients with bile duct obstruction [47,48].

The current study also found alterations in other key metabolites such as ethanol, asparagine, phenylalanine, threonine, and pyruvate, implicated in tumorigenesis. For example, multiple studies have also implicated pyruvate metabolism in tumor progression in GBC patients [49,50]. Furthermore, elevated levels of essential amino acids, such as phenylalanine and threonine, have been reported in hepatocellular carcinoma, where they predict early recurrence and future risk [51,52]. In another study, increased asparagine metabolism was associated with poorer prognosis in patients with hepatocellular carcinoma [33]. Additionally, our study also demonstrated a significant reduction of ethanol in GBC patients compared to BBP patients, independent of the level of bilirubin. These dysregulations may be influenced by exogenous alcohol intake or endogenous ethanol production linked to dysbiosis [53,54,55]. However, the precise etiology of the reduced ethanol concentration observed in GBC patients remains uncertain and warrants further investigation.

Lack of early-stage data limits insights into early tumor biology. Hence, it limits the study’s use in early detection, risk stratification, or monitoring disease progression. Over-representation of late-stage disease could also affect biomarker specificity. The small number of patients recruited for this study could also be a limitation. In addition, the presence of bile stasis and obstructive jaundice in advanced GBC could mask the identification of markers of malignancy. Furthermore, although several findings were identified, the study is descriptive. Future studies would aim to validate these findings in a larger patient cohort.

## 5. Conclusions

The study showed the metabolomic and lipidomic profiles in GBC patients compared to the control group. Demonstrating these profiles enhances our understanding of GBC pathophysiology within the patient population, including identifying potential biomarkers. Future studies with a larger sample size, which should include patients with early disease stages, are critical to investigating the use of the identified markers as diagnostic and prognostic biomarkers. In addition, more studies are needed to explore the mechanistic underpinnings of lipid metabolism dysregulation in GBC.

## Figures and Tables

**Table 1:** Clinicopathological features of gall bladder cancer and benign biliary pathology control patients.

Feature	Control (BBP) (*n* = 27)	GBC (*n* = 40)	*p*-Value
Age (year), median [IQR]	53 [42, 66]	61.5 [55.75 72]	0.0795
Gender			0.610
Female, *n* (%)	18 (66.7)	23 (57.5)	
Male, *n* (%)	9 (33.3)	17 (42.5)	
Tbili (μmol/L), median [IQR]	16.5 [12, 31]	216.5 [93.5, 322.75]	<0.001
Conj Bili (μmol/L), median [IQR]	13.5 [4.75, 26]	174.5 [76.25, 252.75]	<0.001
ALP (U/L), median [IQR]	308.5 [127.25, 499.25]	564 [323.25, 909.25]	0.0396
GGT (U/L), median [IQR]	452 [159, 612]	490 [234, 693.5]	0.436
CRP (mg/L), median [IQR]	22.5 [7.25, 83.25]	74 [40, 191]	0.0234

Abbreviations: ALP, alkaline phosphatase; Conj Bili, conjugated bilirubin; CRP, C-reactive protein; GBC, gall bladder cancer; GGT, gamma-glutamyl transferase; IQR, interquartile range; Tbili, total bilirubin.

**Table 2:** Comparison of lipoprotein analyses in benign biliary pathology and gall bladder cancer patients.

Feature	Control (BBP) Median [IQR]	GBC Median [IQR]	*p*-Value	FDR
VLDL-C (nmol/L)	16.8 [8.4, 21.1]	17.4 [9.8, 26.6]	0.356	0.431
IDL-C (nmol/L)	16.7 [10.0, 23.1]	31.4 [15.6, 54.0]	0.004	0.016
LDL-C (nmol/L)	128.2 [111.1, 151.9]	135.1 [108.1, 158.0]	0.596	0.623
HDL-C (nmol/L)	45.0 [31.9, 61.1]	22.0 [2.3, 43.2]	0.003	0.014
VLDL-TG (nmol/L)	58.2 [41.0, 80.6]	72.5 [54.6, 103.6]	0.122	0.227
IDL-TG (nmol/L)	16.5 [10.4, 19.5]	21.8 [14.0, 37.0]	0.008	0.028
LDL-TG (nmol/L)	23.7 [15.8, 30.7]	33.5 [21.7, 54.6]	0.003	0.014
HDL-TG (nmol/L)	16.7 [13.2, 22.2]	18.8 [14.1, 23.9]	0.469	0.539
VLDL-P (nmol/L)	43.8 [29.8, 59.3]	51.9 [38.2, 74.8]	0.138	0.227
Large VLDL-P (nmol/L)	0.99 [0.77, 1.38]	1.29 [0.90, 1.62]	0.158	0.227
Medium VLDL-P (nmol/L)	4.6 [3.6, 6.2]	5.1 [3.6, 6.6]	0.699	0.699
Small VLDL-P (nmol/L)	37.0 [26.2, 52.9]	47.7 [32.2, 65.6]	0.144	0.227
LDL-P (nmol/L)	1342.6 [1108.7, 1514.3]	1507.9 [1181.1, 1871.1]	0.087	0.199
Large LDL-P (nmol/L)	211.9 [175.1, 248.5]	229.5 [165.5, 280.7]	0.341	0.431
Medium LDL-P (nmol/L)	457.9 [325.7, 610.4]	657.1 [429.20, 841.0]	0.031	0.079
Small LDL-P (nmol/L)	642.8 [547.5, 731.1]	668.1 [558.7, 754.1]	0.554	0.607
HDL-P (mol/L)	22.7 [13.7, 31.1]	13.2 [5.2, 22.2]	0.002	0.014
Large HDL-P (mol/L)	0.3 [0.3, 0.3]	0.3 [0.2, 0.3]	0.141	0.227
Medium HDL-P (mol/L)	10.0 [9.5, 12.2]	8.7 [6.3, 11.4]	0.013	0.039
Small HDL-P (mol/L)	12.3 [2.4, 20.2]	4.1 [0.1, 10.0]	0.003	0.014
VLDL-Z (nm)	42.2 [42.2, 42.2]	42.2 [42.2, 42.2]	0.334	0.431
LDL-Z (nm)	21.3 [21.2, 21.5]	21.4 [21.2, 21.6]	0.155	0.227
HDL-Z (nm)	8.4 [8.3, 8.9]	8.8 [8.5, 9.6]	0.003	0.014

Abbreviations: C, cholesterol; GBC, gall bladder cancer; FDR, false discovery rate; HDL, high-density lipoprotein; IQR, interquartile range; LDL, low-density lipoprotein; P, particle; TG, triglycerides; VLDL, very-low-density lipoprotein.

**Table 3:** Comparison of metabolite concentrations in benign biliary pancreatitis controls and gall bladder cancer patients.

Feature	BBP (Median [IQR])	GBC (Median [IQR])	*p*-Value	FDR
Formate	0.01 [0.01, 0.02]	0.02 [0.0,1 0.02]	0.499	0.713
Phenylalanine	0.14 [0.09, 0.23]	0.26 [0.14, 0.33]	0.013	0.276
Tyrosine	0.08 [0.05, 0.12]	0.08 [0.04, 0.12]	0.828	0.920
Unknown signal at 7.14 ppm	0 [0, 0.02]	0.01 [0, 0.10]	0.044	0.276
Histidine	0.07 [0.03, 0.09]	0.06 [0.02, 0.08]	0.138	0.459
Urea	0.26 [0.11, 0.45]	0.25 [0.15, 0.34]	0.894	0.932
Glucose	1.89 [1.40, 2.40]	1.71 [0.91, 2.34]	0.579	0.762
Mannose	0.04 [0.02, 0.06]	0.04 [0.02, 0.06]	0.933	0.942
Ascorbate	0.01 [0, 0.01]	0 [0, 0.004]	0.243	0.534
Lactose	0.02 [0.01, 0.03]	0.02 [0.01, 0.03]	0.476	0.700
Lactate	1.64 [0.80, 2.19]	1.44 [0.91, 2.38]	0.942	0.942
Creatinine	0.11 [0.05, 0.13]	0.12 [0.07, 0.15]	0.278	0.534
Creatine	0.03 [0.02, 0.06]	0.06 [0.02, 0.09]	0.162	0.475
Glycerol	0.27 [0.12, 0.33]	0.23 [0.08, 0.36]	0.625	0.765
Threonine	0.18 [0.10, 0.24]	0.10 [0.07, 0.15]	0.021	0.276
Glycine	0.72 [0.58, 0.92]	0.58 [0.27, 0.76]	0.038	0.276
Proline	0.12 [0.02, 0.22]	0.06 [0.02, 0.14]	0.149	0.464
Methanol	0.06 [0.04, 0.09]	0.05 [0.02, 0.10]	0.419	0.654
Asparagine	0 [0, 0.01]	0.006 [0, 0.02]	0.022	0.276
N,N-dimethylglycine	0.02 [0.01, 0.04]	0.03 [0.01, 0.05]	0.278	0.534
Citrate	0.09 [0.02, 0.23]	0.04 [0, 0.16]	0.267	0.534
Glutamine	0.34 [0.22, 0.51]	0.33 [0.14, 0.54]	0.642	0.765
Pyruvate	0.06 [0.03, 0.1]	0.11 [0.04, 0.19]	0.030	0.276
Glutamate	0.42 [0.27, 0.81]	0.39 [0.21, 0.73]	0.530	0.717
Acetoacetate	0.13 [0.08, 0.20]	0.1 [0.05, 0.20]	0.334	0.567
Lysine	0.02 [0.01, 0.03]	0.02 [0.01, 0.03]	0.847	0.921
Acetate	0.10 [0.06, 0.13]	0.09 [0.05, 0.13]	0.523	0.717
Alanine	1.06 [0.39, 1.30]	0.78 [0.38, 1.08]	0.197	0.534
2-hydroxyisobutyrate	0.01 [0.002, 0.02]	0.01 [0.004, 0.02]	0.340	0.567
3-hydroxybutyrate	0.18 [0.01, 0.68]	0.19 [0.11, 0.62]	0.299	0.534
Ethanol	0.55 [0.13, 1.23]	0 [0, 0.31]	<0.001	0.033
Isopropanol	0.07 [0.001, 0.27]	0 [0, 0.13]	0.073	0.367
Propylene glycol	0.01 [0, 0.02]	0.01 [0, 0.03]	0.622	0.765
Valine	0.38 [0.23, 0.50]	0.43 [0.17, 0.52]	0.791	0.919
Isoleucine	0.1 [0.03, 0.14]	0.04 [0.01, 0.11]	0.133	0.459
Leucine	0.45 [0.25, 0.67]	0.41 [0.19, 0.54]	0.252	0.534
2-hydroxybutyrate	0.03 [0, 0.05]	0.05 [0.01, 0.09]	0.095	0.432
Protein NH	130.2 [59.4, 160.1]	123.4 [58.3, 143.2]	0.440	0.667
Unsaturated lipid (-CH=CH-)	17.08 [9.57, 31.18]	19.79 [10.97, 27.96]	0.819	0.920
Lipid (alpha-CH_2_)	3.06 [1.34, 4.74]	3.42 [1.61, 8.74]	0.214	0.534
Cholesterol backbone (-C(18)H_3_),	2.69 [1.89, 3.53]	1.62 [0.79, 2.90]	0.039	0.276
Lipid (=CH-CH_2_-CH=)	10.42 [5.84, 14.19]	8.68 [4.55, 11.11]	0.205	0.534
Glycerol phospholipid	0.29 [0.12, 0.68]	0.52 [0.16, 1.26]	0.068	0.367
Phospholipid	4.07 [2.53, 5.14]	3.24 [1.54, 4.56]	0.111	0.459
Lipid (beta-CH_2_)	15.39 [10.21, 17.51]	11.89 [6.02, 19.75]	0.385	0.621
Lipid (-(-CH_2_-)*_n_*-)	104.3 [45.9, 159.0]	126.3 [59.8, 188.3]	0.294	0.534
Lipid (-CH_3_-)	77.77 [34.58, 96.66]	71.65 [32.79, 99.77]	0.875	0.931
GlycB	0.89 [0.48, 1.27]	1.12 [0.75, 1.50]	0.138	0.459
GlycA	4.51 [3.21, 5.69]	4.63 [2.58, 7.11]	0.629	0.765

Abbreviations: FDR, false discovery rate; GBC, gall bladder cancer; ppm, parts per million.

## Data Availability

All data produced in the present study are available upon reasonable request to the authors.

## Abbreviations

The following abbreviations are used in this manuscript:

^1^H	Proton
95%CI	95% confidence interval
ALP	Alkaline phosphatase
AUC	Area under the curve
BBP	Benign biliary pathology
C	Cholesterol
CE	Cholesterol ester
Conj Bili	Conjugated bilirubin
CRP	C-reactive protein
DIFF	Standard diffusion-edited
FC	Free cholesterol
FDR	False discovery rate
GBC	Gall bladder cancer
GGT	Gamma-glutamyl transferase
HDL	High-density lipoprotein
IDL	Intermediate-density lipoprotein
IQR	Interquartile range
LDL	Low-density lipoprotein
NMR	Nuclear magnetic resonance
NOESY	Nuclear Overhauser effect spectroscopy
OR	Odds ratio
P	Particle number
PPM	Parts per million
ROC	Receiver operating curve
S	Size
Tbili	Total bilirubin
TG	Triglycerides
VLDL	Very-low-density lipoprotein

## References

[1] Bray F., Laversanne M., Sung H., Ferlay J., Siegel R.L., Soerjomataram I., Jemal A. (2024). Global cancer statistics 2022: GLOBOCAN estimates of incidence and mortality worldwide for 36 cancers in 185 countries. CA A Cancer J. Clin..

[2] Torre L.A., Siegel R.L., Islami F., Bray F., Jemal A. (2018). Worldwide burden of and trends in mortality from gallbladder and other biliary tract cancers. Clin. Gastroenterol. Hepatol..

[3] Abdu S.M., Assefa E.M. (2025). Prevalence of gallstone disease in Africa: A systematic review and meta-analysis. BMJ Open Gastroenterol..

[4] Khan Z.A., Khan M.U., Brand M. (2022). Gallbladder cancer in Africa: A higher than expected rate in a “low-risk” population. Surgery.

[5] Rawla P., Sunkara T., Thandra K.C., Barsouk A. (2019). Epidemiology of gallbladder cancer. Clin. Exp. Hepatol..

[6] Tirca L., Savin C., Stroescu C., Balescu I., Petrea S., Diaconu C., Gaspar B., Pop L., Varlas V., Hasegan A. (2024). Risk factors and prognostic factors in GBC. J. Clin. Med..

[7] Zhou Y., Yuan K., Yang Y., Ji Z., Zhou D., Ouyang J., Wang Z., Wang F., Liu C., Li Q. (2023). Gallbladder cancer: Current and future treatment options. Front. Pharmacol..

[8] Hundal R., Shaffer E.A. (2014). Gallbladder cancer: Epidemiology and outcome. Clin. Epidemiol..

[9] Schmidt M.A., Marcano-Bonilla L., Roberts L.R. (2019). Gallbladder cancer: Epidemiology and genetic risk associations. Chin. Clin. Oncol..

[10] Raza S.A., da Costa W.L., Thrift A.P. (2022). Increasing incidence of gallbladder cancer among non-Hispanic Blacks in the United States: A birth cohort phenomenon. Cancer Epidemiol. Biomark. Prev..

[11] Baichan P., Naicker P., Augustine T.N., Smith M., Candy G., Devar J., Nweke E.E. (2023). Proteomic analysis identifies dysregulated proteins and associated molecular pathways in a cohort of gallbladder cancer patients of African ancestry. Clin. Proteom..

[12] Cacciatore S., Wium M., Licari C., Ajayi-Smith A., Masieri L., Anderson C., Salukazana A.S., Kaestner L., Carini M., Carbone G.M. (2021). Inflammatory metabolic profile of South African patients with prostate cancer. Cancer Metab..

[13] Mazibuko J., Elebo N., Williams A.A., Omoshoro-Jones J., Devar J.W., Smith M., Cacciatore S., Fru P.N. (2024). Metabolites and lipoproteins may predict the severity of early Acute Pancreatitis in a South African cohort. Biomedicines.

[14] Elebo N., Omoshoro-Jones J., Fru P.N., Devar J., De Wet van Zyl C., Vorster B.C., Smith M., Cacciatore S., Zerbini L.F., Candy G. (2021). Serum metabolomic and lipoprotein profiling of pancreatic ductal adenocarcinoma patients of african ancestry. Metabolites.

[15] Elia I., Haigis M.C. (2021). Metabolites and the tumour microenvironment: From cellular mechanisms to systemic metabolism. Nat. Metab..

[16] Cacciatore S., Loda M. (2015). Innovation in metabolomics to improve personalized healthcare. Ann. N. Y. Acad. Sci..

[17] Du Y., Wijaya W.A., Liu W.H. (2024). Advancements in metabolomics research in benign gallbladder diseases: A review. Medicine.

[18] Sonkar K., Behari A., Kapoor V., Sinha N. (2013). 1H NMR metabolic profiling of human serum associated with benign and malignant gallstone diseases. Metabolomics.

[19] Alam M.S., Harun-Ar-Rashid A., Islam M.N., Jannat F. (2021). The Association of the Serum Lipid Abnormalities in Cholelithiasis Patients. Sch. J. Appl. Med. Sci..

[20] Hayat S., Hassan Z., Changazi S.H., Zahra A., Noman M., ul Abdin M.Z., Javed H., Ans A.H. (2019). Comparative analysis of serum lipid profiles in patients with and without gallstones: A prospective cross-sectional study. Ann. Med. Surg..

[21] El Moghazy W., Kashkoush S., Meeberg G., Kneteman N. (2016). Incidence, characteristics, and prognosis of incidentally discovered hepatocellular carcinoma after liver transplantation. J. Transplant..

[22] Perez P., Rodriguez-Peralvarez M., Guerrero L., Gonzalez V., Sanchez R., Centeno M., Poyato A., Briceno J., Sanchez-Frias M., Montero J.L. (2017). Incidental hepatocellular carcinoma after liver transplantation: Prevalence, histopathological features and prognostic impact. PLoS ONE.

[23] Hara T., Eguchi S., Yoshizumi T., Akamatsu N., Kaido T., Hamada T., Takamura H., Shimamura T., Umeda Y., Shinoda M. (2021). Incidental intrahepatic cholangiocarcinoma in patients undergoing liver transplantation: A multi-center study in Japan. J. Hepato-Biliary-Pancreat. Sci..

[24] Schwenk L., Rohland O., Ali-Deeb A., Dondorf F., Settmacher U., Rauchfuß F. (2023). Liver Transplantation for Incidental Cholangiocarcinoma or Combined Hepatocellular Carcinoma/Cholangiocarcinoma—Own Experiences and Review of the Literature. Cancers.

[25] Serkova N., Fuller T.F., Klawitter J., Freise C.E., Niemann C.U. (2005). 1H-NMR–based metabolic signatures of mild and severe ischemia/reperfusion injury in rat kidney transplants. Kidney Int..

[26] Otvos J.D., Shalaurova I., Wolak-Dinsmore J., Connelly M.A., Mackey R.H., Stein J.H., Tracy R.P. (2015). GlycA: A composite nuclear magnetic resonance biomarker of systemic inflammation. Clin. Chem..

[27] Mallol R., Amigó N., Rodríguez M.A., Heras M., Vinaixa M., Plana N., Rock E., Ribalta J., Yanes O., Masana L. (2015). Liposcale: A novel advanced lipoprotein test based on 2D diffusion-ordered 1H NMR spectroscopy. J. Lipid Res..

[28] Jeyarajah E.J., Cromwell W.C., Otvos J.D. (2006). Lipoprotein particle analysis by nuclear magnetic resonance spectroscopy. Clin. Lab. Med..

[29] Rodríguez-Tomàs E., Murcia M., Arenas M., Arguís M., Gil M., Amigó N., Correig X., Torres L., Sabater S., Baiges-Gayà G. (2019). Serum paraoxonase-1-related variables and lipoprotein profile in patients with lung or head and neck cancer: Effect of radiotherapy. Antioxidants.

[30] Abdel-Shafy E.A., Kassim M., Vignoli A., Mamdouh F., Tyekucheva S., Ahmed D., Ojo D., Price B., Socciarelli F., Duarte-Delgado N.P. (2025). KODAMA enables self-guided weakly supervised learning in spatial transcriptomics. bioRxiv.

[31] Cacciatore S., Luchinat C., Tenori L. (2014). Knowledge discovery by accuracy maximization. Proc. Natl. Acad. Sci. USA.

[32] Cacciatore S., Tenori L., Luchinat C., Bennett P.R., MacIntyre D.A. (2017). KODAMA: An R package for knowledge discovery and data mining. Bioinformatics.

[33] Bai J., Tang R., Zhou K., Chang J., Wang H., Zhang Q., Shi J., Sun C. (2022). An asparagine metabolism-based classification reveals the metabolic and immune heterogeneity of hepatocellular carcinoma. BMC Med. Genom..

[34] Ong S.L., Garcea G., Thomasset S.C., Neal C.P., Lloyd D.M., Berry D.P., Dennison A.R. (2008). Ten-year experience in the management of gallbladder cancer from a single hepatobiliary and pancreatic centre with review of the literature. HPB.

[35] Barahona Ponce C., Scherer D., Brinster R., Boekstegers F., Marcelain K., Gárate-Calderón V., Müller B., De Toro G., Retamales J., Barajas O. (2021). Gallstones, body mass index, C-Reactive protein, and gallbladder cancer: Mendelian randomization analysis of Chilean and European genotype data. Hepatology.

[36] Liu Z., Kemp T.J., Gao Y.-T., Corbel A., McGee E.E., Roa J.C., Wang B., Araya J.C., Shen M.-C., Rashid A. (2018). Circulating levels of inflammatory proteins and survival in patients with gallbladder cancer. Sci. Rep..

[37] Gomez-Rosas P., Tartari C.J., Russo L., Bolognini S., Ticozzi C., Romeo D., Schieppati F., Barcella L., Falanga A., Marchetti M. (2025). Thromboinflammatory Biomarkers Are Early Predictors of Disease Progression in Non-Small Cell Lung Cancer Patients. Cancers.

[38] Kaczmarek F., Marcinkowska-Gapińska A., Bartkowiak-Wieczorek J., Nowak M., Kmiecik M., Brzezińska K., Dotka M., Brosz P., Firlej W., Wojtyła-Buciora P. (2025). Blood-Based Biomarkers as Predictive and Prognostic Factors in Immunotherapy-Treated Patients with Solid Tumors—Currents and Perspectives. Cancers.

[39] Bruserud Ø., Aarstad H.H., Tvedt T.H.A. (2020). Combined C-reactive protein and novel inflammatory parameters as a predictor in cancer—What can we learn from the hematological experience?. Cancers.

[40] Maran L., Hamid A., Hamid S.B.S. (2021). Lipoproteins as markers for monitoring cancer progression. J. Lipids.

[41] Delmas D., Mialhe A., Cotte A.K., Connat J.-L., Bouyer F., Hermetet F., Aires V. (2025). Lipid metabolism in cancer: Exploring phospholipids as potential biomarkers. Biomed. Pharmacother..

[42] Giacomini I., Gianfanti F., Desbats M.A., Orso G., Berretta M., Prayer-Galetti T., Ragazzi E., Cocetta V. (2021). Cholesterol metabolic reprogramming in cancer and its pharmacological modulation as therapeutic strategy. Front. Oncol..

[43] Lee-Rueckert M., Jauhiainen M., Kovanen P.T., Escolà-Gil J.C. (2025). Lipids and lipoproteins in the interstitial tissue fluid regulate the formation of dysfunctional tissue-resident macrophages: Implications for atherogenic, tumorigenic, and obesogenic processes. Semin. Cancer Biol..

[44] Madaudo C., Bono G., Ortello A., Astuti G., Mingoia G., Galassi A.R., Sucato V. (2024). Dysfunctional high-density lipoprotein cholesterol and coronary artery disease: A narrative review. J. Pers. Med..

[45] Ashorobi D., Liao H. (2024). Lipoprotein X-Induced Hyperlipidemia. StatPearls [Internet].

[46] Crook M. (2013). Lipoprotein X: Clinical implications. Ann. Clin. Biochem..

[47] Heimerl S., Boettcher A., Kaul H., Liebisch G. (2016). Lipid profiling of lipoprotein X: Implications for dyslipidemia in cholestasis. Biochim. Biophys. Acta BBA Mol. Cell Biol. Lipids.

[48] Phatlhane D.V., Zemlin A.E. (2016). Severe hypercholesterolemia mediated by lipoprotein X in a patient with cholestasis. Ann. Hepatol..

[49] Liang R.-P., Zhang X.-X., Zhao J., Zhu R.-T., Wang W.-J., Lu Q.-W., Sun Y.-L. (2022). Ubiquitin-specific protease 3 facilitates cell proliferation by deubiquitinating pyruvate kinase L/R in gallbladder cancer. Lab. Investig..

[50] Ma M.-z., Zhang Y., Weng M.-z., Wang S.-h., Hu Y., Hou Z.-y., Qin Y.-y., Gong W., Zhang Y.-J., Kong X. (2016). Long noncoding RNA GCASPC, a target of miR-17-3p, negatively regulates pyruvate carboxylase–dependent cell proliferation in gallbladder cancer. Cancer Res..

[51] Morine Y., Utsunomiya T., Yamanaka-Okumura H., Saito Y., Yamada S., Ikemoto T., Imura S., Kinoshita S., Hirayama A., Tanaka Y. (2022). Essential amino acids as diagnostic biomarkers of hepatocellular carcinoma based on metabolic analysis. Oncotarget.

[52] Assi N., Fages A., Vineis P., Chadeau-Hyam M., Stepien M., Duarte-Salles T., Byrnes G., Boumaza H., Knueppel S., Kuehn T. (2015). A statistical framework to model the meeting-in-the-middle principle using metabolomic data: Application to hepatocellular carcinoma in the EPIC study. Mutagenesis.

[53] Mitchell M.C., Teigen E.L., Ramchandani V.A. (2014). Absorption and peak blood alcohol concentration after drinking beer, wine, or spirits. Alcohol Clin. Exp. Res..

[54] Mbaye B., Borentain P., Magdy Wasfy R., Alou M.T., Armstrong N., Mottola G., Meddeb L., Ranque S., Gérolami R., Million M. (2022). Endogenous ethanol and triglyceride production by gut Pichia kudriavzevii, Candida albicans and Candida glabrata yeasts in non-alcoholic steatohepatitis. Cells.

[55] Guo G.-J., Yao F., Lu W.-P., Xu H.-M. (2023). Gut microbiome and metabolic-associated fatty liver disease: Current status and potential applications. World J. Hepatol..

